# In Situ Laser Fenestration Technique: Bench-Testing of Aortic Endograft to Guide Clinical Practice

**DOI:** 10.1177/15266028221119315

**Published:** 2022-08-24

**Authors:** Matthew Joe Grima, Anders Wanhainen, David Lindström

**Affiliations:** 1 Department of Surgical Sciences, Section of Vascular Surgery, Uppsala University, Uppsala, Sweden; 2 Faculty of Medicine and Surgery, University of Malta, L-iMsida, Malta; 3 Department of General Surgery, Vascular Unit, Mater Dei Hospital, L-iMsida, Malta; 4 Department of Surgical and Perioperative Sciences, Surgery, Umeå University, Umeå, Sweden

**Keywords:** aorta, laser, stents, fenestration, endovascular procedures

## Abstract

**Purpose:**

In situ laser fenestration (ISLF) is a recently introduced technology that offers the potential to perform total endovascular treatment of aortic arch and thoracoabdominal aortic pathologies in the acute setting. This experiment’s aim was to assess ISLF in some currently common aortic endografts and bridging stent-grafts.

**Materials and Methods:**

Three different aortic endografts were evaluated: (1) Zenith Alpha, (2) Zenith TX2, and (3) Conformable GORE TAG. Each endograft was submerged in 37°C saline to create fenestrations using the 308 nm CVX-300 Excimer Laser System fitted with a 2.3 mm diameter Turbo-Elite laser atherectomy catheter compatible with a 0.018″ guidewire. Three different 8 mm bridging stent-grafts were evaluated: (1) BeGraft peripheral, (2) BeGraft peripheral plus, and (3) GORE VIABAHN VBX Balloon Expandable. All bridging stent-grafts were deployed and exposed to different balloon sizes and pressures. The ISLFs and bridging stent-grafts were then evaluated for any tears, stenoses, and seal.

**Results:**

A laser fenestration was consistently rapidly obtained in the Zenith Alpha and the Zenith TX2 endografts while it proved difficult to achieve a timely fenestration in the C-TAG. No fabric tears were noted in the Zenith Alpha and Zenith TX2 when inflating Armada (Abbott) 8 mm balloon in the fenestrations with pressures up to 15 atmospheres (rated burst pressure) nor when flaring bridging stent-grafts with balloons up to 12 mm in diameter at 10 atmospheres, while major tears were frequently noted in the C-TAG when the Armada 8 mm balloons were inflated. BeGraft Peripheral and BeGraft Peripheral Plus were all firmly attached to the fenestrations showing good seal on manual testing, while every sixth VBX bridging stent-graft displayed poorer attachment to the fenestration before dilatation at high pressure. Commonly, significant stenoses remained in the bridging stent-grafts after dilatation at nominal pressure, which could only be eradicated with high-pressure balloons.

**Conclusion:**

In this limited bench-test, Dacron endografts responded well to the ISLF technology. Satisfactory deployment of the bridging stent was noted only after inflation and/or flaring with high-pressure balloons. Further work with different types of commercially-available bridging stent-grafts and endografts to assess the durability of in situ fenestration (ISF) and bridging stents in ISF is recommended.

**Clinical Impact:**

This report on experimental in situ laser fenestration provide important insights for clinicians considering using in situ laser fenestration of aortic stentgrafts in vivo. In particular, different laser settings were tested together with a selection of aortic stentgrafts. Also, the target pressure needed in PTA balloons to dilate the fenestrations and any subsequent tears in the fabric were noted. This was followed by deployment of assorted balloon-expandable stentgrafts with estimation of residual stenosis and seal.

## Introduction

Custom-built branched and fenestrated endografts to treat patients with complex aortic pathologies have been used for 2 decades.^1,2^ However, these devices require weeks or months to be produced.^3,4^ Off-the shelf availability is limited to parallel-grafts^5^ and non-customized branched endografts.^6,7^ The use of a needle, or a radiofrequency probe, or a laser catheter to create in situ fenestration (ISF) through the endograft^8^ has recently emerged and offers the potential to perform total endovascular treatment in aortic arch and thoracoabdominal aorta in the emergency setting. The technique of ISF consists of 3 separate methods: first, creation of a fenestration in the fabric at the level of the target vessel, followed by dilatation to the appropriate size using noncompliant balloons, and finally antegrade or retrograde stenting of the target vessel using bridging stent-grafts.^9,10^

Among the 3 methods available for ISF in aortic endografts, there is limited evidence to support the choice of one technique over another.^11^ Laser fenestration is, however, becoming the most widely-accepted method.^10,11^ A potential limitation with the in situ laser fenestration (ISLF) method in general, and as an energy-based technology, is the risk of fraying the fabrics during balloon dilation.^12^ A recent review found that dilatation should be done with standard noncompliant balloons and not exceed 8 mm in diameter.^13^

In 2020, a laser system (Philips Excimer CVX-300) was introduced at the authors’ University Hospital to provide endovascular treatment for patients who presented with acute aortic pathology not suitable for open surgery. Given the limited evidence on optimal laser technique, the aim of our study was to assess how the aortic endografts respond to laser fenestrations and how the bridging stent-grafts will behave in the ISF created using laser technology.

## Materials and Methods

In vitro testing of ISLF of aortic endografts and deployment of bridging stent-grafts in the fenestrations

Three different commercially-available thoracic aortic stent-grafts were used to test the laser fenestration technique: Zenith Alpha, Zenith TX2 (Cook Medical LLC, Bloomington, In, USA), and Conformable GORE TAG (C-TAG, W.L. Gore & Associates, Inc, Flagstaff, Arizona, USA). Each aortic stent-graft was submerged in 37°C saline to create fenestrations using the 308 nm CVX-300 Excimer Laser System (Spectranetics, Colorado Springs, CO, USA) (Supplementary Figure 1 and Supplementary Video 1). The laser system was fitted with 2.3 mm diameter, 150 cm long Turbo-Elite Laser Atherectomy Catheter which is compatible with a 0.018″ guidewire. Three different experiments were performed:

I. To assess the time (in seconds) required to make a fenestration in each of the grafts using different fluency strengths (mJ/mm^2^) and pulse rates (pulses/s) in the laser. The different settings were as follows:
  Fluency 30 mJ/mm^2^ and rate 25 pulses/s
  Fluency 45 mJ/mm^2^ and rate 25 pulses/s
  Fluency 45 mJ/mm^2^ and rate 60 pulses/s
  Fluency 60 mJ/mm^2^ and rate 60 pulses/s
  Fluency 60 mJ/mm^2^ and rate 80 pulses/s

For each setting, 10 fenestrations in each graft were applied.

II. To assess the integrity of the aortic stent-graft fabric when the fenestration is subjected to balloon dilatation.

Ten fenestrations were applied to each graft. Each fenestration was predilated with an Armada 6 mm balloon at nominal pressure of 8 atmospheres followed by gradual dilatation with an Armada 8 mm balloon; first, at nominal pressure of 8 atmospheres and then at rated burst pressure of 15 atmospheres. This was done to mimic the ISLF technique for the visceral vessels. The fenestrations and aortic stent-grafts were assessed after each step for any tears in the fabric.

III. To assess the behavior of the bridging stent-grafts when deployed in the laser fenestrations.

For each of the 3 different bridging stent-grafts to be evaluated, ISLF were created in the 3 different aortic stent-grafts, which were first predilated with an Armada 6 mm balloon at nominal pressure of 8 atmospheres for 30 seconds. A total of 9 (3 for each aortic stent-graft) 8 mm GORE VIABAHN VBX Balloon Expandable Endoprosthesis (VBX bridging stent-graft) were deployed at nominal pressure of 12 atmospheres, 20 (10 for Zenith Alpha and 10 for Zenith TX2) 8 mm BeGraft Peripheral (Bentley InnoMed GmbH) deployed at nominal pressure 8 atmospheres, and 20 (10 for Zenith Alpha and 10 for Zenith TX2) 8 mm BeGraft Peripheral Plus (Bentley InnoMed GmbH) deployed at nominal pressure 9 atmospheres. Each bridging stent-graft was thereafter gradually flared, with an Armada 10 mm balloon at nominal pressure of 6 atmospheres to mimic renal artery stenting, followed by an Armada 12 mm balloon at nominal pressure of 4 atmospheres to mimic a superior mesenteric artery stenting, and finally with an Armada 12 mm balloon at 10 atmospheres to simulate a higher pressure balloon. After following this experiment protocol, any remaining stenoses in the bridging stent-grafts were addressed by flaring the stent with an Armada 8 mm balloon up to 15 atmospheres rated burst pressure, Armada 10 mm balloon up to 13 atmospheres rated burst pressure and a Atlas GOLD (BD) 12 mm balloon up to 18 atmospheres rated burst pressure, respectively. After each step, the bridging stent-grafts and ISLF were assessed by means of ocular examination for the presence of any fabric tear or stenosis—graded as (1) no stenosis, (2) minimal stenosis, and (3) significant stenosis—and by manual examination for evaluation of the attachment of the bridging stent-grafts to the fenestrations.

### Statistical Analysis

For the first experiment, the time (in seconds) taken to create the fenestrations in each graft was assessed for normality using the Shapiro-Wilk test. For normal distributed data, the mean and standard deviation (SD) were analyzed. Statistical analysis was carried out using GraphPad Prism version 9.2.0 for MacOS, GraphPad Software, San Diego, California, USA, www.graphpad.com.

## Results

### Experiment I: Time (in Seconds) Required to Make a Fenestration Using a Variety of Fluency and Pulse Rates

When applying fluency at 60 mJ/mm^2^ and rate at 80 pulses/s, 2 fenestrations in the C-TAG (without lamellae) took 30 and 55 seconds, respectively. Thus, a decision was taken to discontinue with the tests due to safety concerns in clinical practice.

The quickest way to create an ISLF was noted with the Zenith Alpha endograft. This was noted even with the minimal settings of fluency of 30 mJ/mm^2^ and pulse rate of 25 pulses/s. The mean time taken to create a fenestration in Zenith Alpha using these settings was 1.7 seconds (SD 0.2), while 0.8 seconds (SD 0.1) were needed to create a fenestration using fluency of 45 mJ/mm^2^ and pulse rate of 25 pulses/s. It was difficult to time the ISLF using fluency of 45 mJ/mm2 and pulse rate of 60 pulses/s as the fenestrations were created in much less than 0.5 seconds.

In situ laser fenestration in the Zenith TX2 was also easily achieved within a reasonable time frame. At fluency of 30 mJ/mm^2^ and pulse rate of 25 pulses/s, a mean of 1.8 seconds (SD 0.3) was recorded to create an ISLF while at fluency of 45 mJ/mm^2^ and pulse rate of 25 pulses/s, it took 1.3 seconds (SD 0.2). At fluency of 45 mJ/mm2 and pulse rate of 60 pulses/s, an ISLF in Zenith TX2 took a mean of 0.5 seconds (SD 0.1).

Raw data for experiment I are provided in Supplementary Table 1.

### Experiment II: Assessing the Integrity of the Aortic Endograft Fabric When the Fenestration Is Subjected to Balloon Dilatation

**Figure 1.**
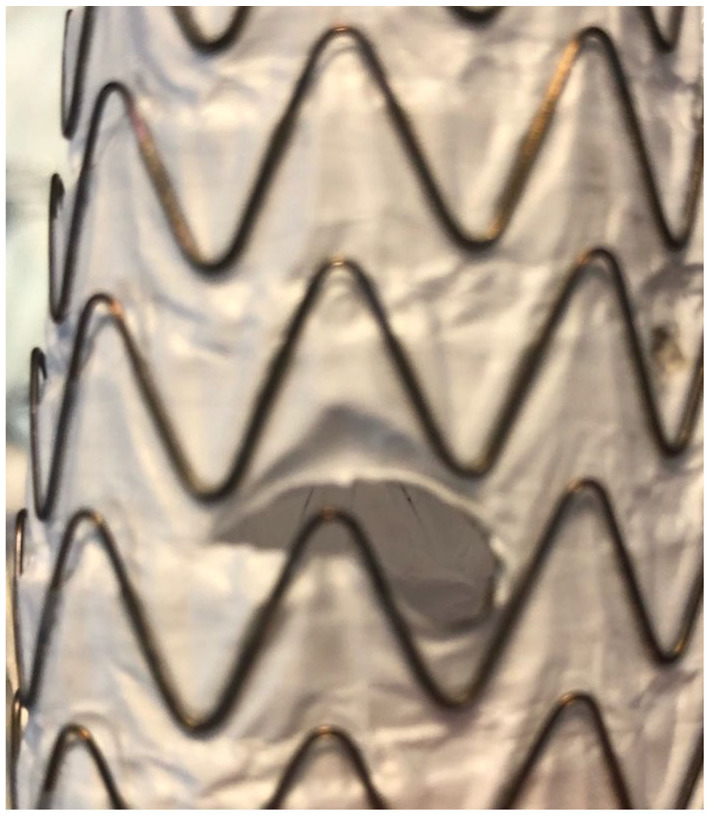
A tear in the C-TAG in the weft direction (perpendicular to the flow direction).

When inflating the Armada 8 mm balloon in the fenestrations in the Zenith Alpha graft and in the Zenith TX2, no fabric tears were noted at 6 and 15 atmospheres, even if the fenestrations were close to the stitch holes. When inflating the Armada 8 mm balloon in the fenestrations in the C-TAG, there was a large fabric tear in 2 fenestrations (out of 10 fenestrations) after 6 atmospheres, and in 5 fenestrations (out of 10 fenestrations) after 15 atmospheres. All tears were in the weft direction (perpendicular to the flow direction) (Figure 1).

### Experiment III: To Assess the Behavior of the Bridging Stent-Grafts Deployed in the Laser Fenestrations

When deploying and flaring 1 of the 3 VBX bridging stent-grafts, a tear was produced in the C-TAG. As the C-TAG was difficult to fenestrate and fabric tears were commonly noted in the endograft after dilating the fenestrations as well, it was considered futile to continue the experiment with the BeGraft Peripheral and BeGraft Peripheral Plus bridging stents in the C-TAG. Overall, there were no tears in the fabric of either the Zenith Alpha or Zenith TX2 endografts or the bridging stent-grafts (VBX, BeGraft peripheral, BeGraft peripheral plus).

BeGraft Peripheral and BeGraft Peripheral Plus were all firmly attached to the fenestrations showing good seal on manual testing, while every sixth VBX bridging stent-graft displayed poorer attachment to the fenestration before dilatation at high pressure.

**Figure 2.**
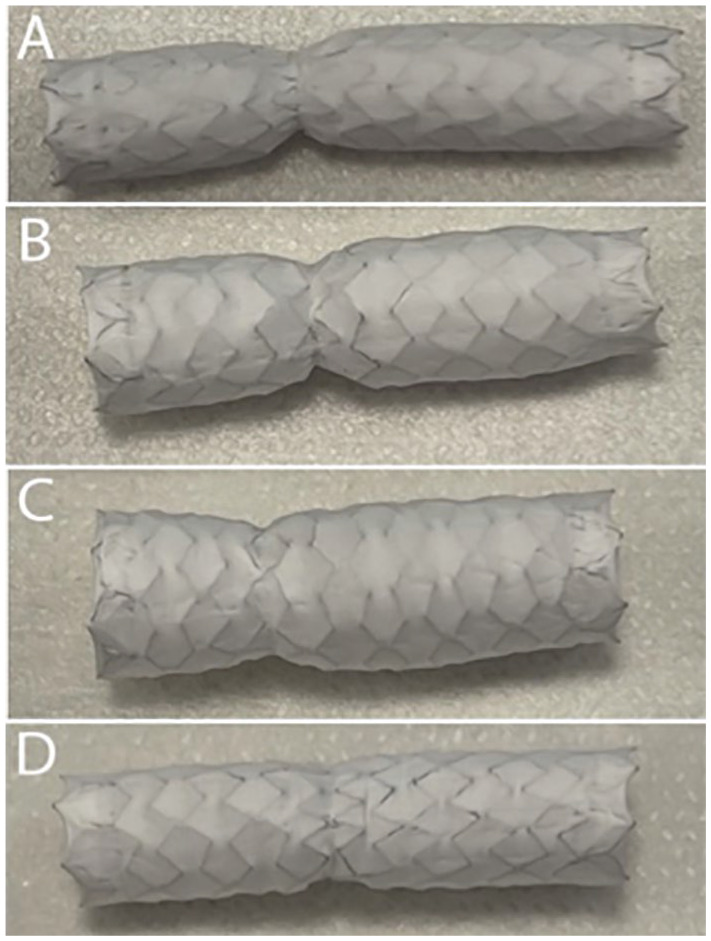
Photo of BeGraft bridging stent-graft 8 × 37 mm removed from laser fenestrations in Zenith Alpha endograft after inflation with different types of plain balloons and the level of stenosis was noted: (A) Expanded with original balloon at nominal pressure (8 atmospheres), 4.5 mm in external diameter (at level of stenosis); significant stenosis. (B) Flared with 10 mm balloon at 6 atmospheres, 5.5 mm in external diameter; significant stenosis. (C) Flared with 12 mm balloon at 4 atmospheres, 7.4 mm in external diameter; minimal stenosis. (D) Expanded with Armada 8 mm balloon at burst pressure of 15 atmospheres, 7.7 mm in external diameter; no stenosis. (This last setting was tried 3 times in a separate Experiment on Zenith Alpha endograft: no spin, no tears, no stenosis were noted.)

**Figure 3.**
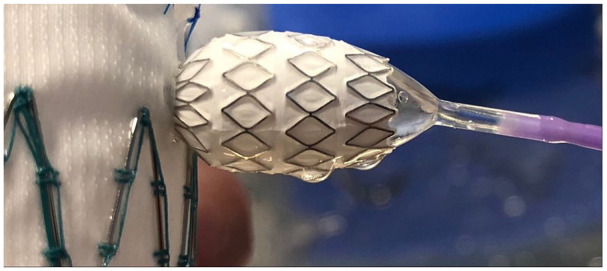
Significant stenosis in the bridging stent-graft.

**Figure 4.**
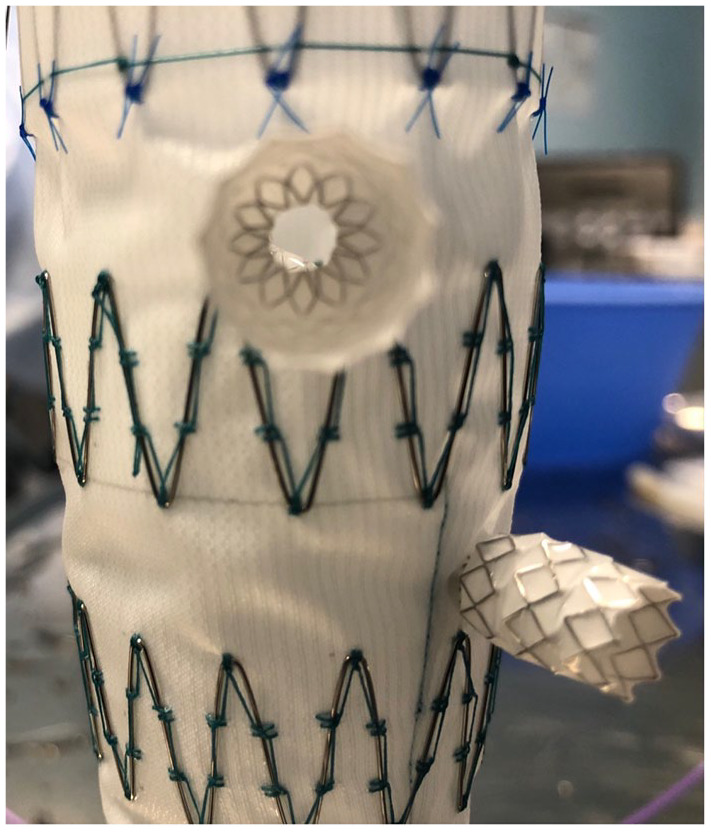
Significant stenosis in the bridging stent-graft.

Commonly, significant stenoses were initially obtained in the bridge stents (Figures 2–4). In the Zenith Alpha endograft, no stenoses was left behind after flaring the stents up to 10 atmospheric pressure irrespective of what bridging stent-graft was used. When VBX stent was deployed in the Zenith TX2, higher pressure Atlas GOLD 12 mm balloon at 11 atmospheres was needed to remove residual stenosis. When deploying BeGraft Peripheral Plus in Zenith TX2, the stenosis was not possible to remove with a maximum of a 12 mm balloon flared at 10 atmospheres. In 5 of these cases, the use of an Armada 8 mm balloon flared up to 15 atmospheres was used to eliminate the stenoses.

Raw data are provided in detail in Supplementary Table 2.

## Discussion

This experimental analysis revealed several interesting facts, which may be of great value in clinical practice. The quickest way to create an ISLF was noted with the Zenith Alpha endograft. This was noted with the minimal settings of fluency of 45 mJ/mm^2^ and pulse rate of 25 pulses/s. Increasing the pulse rate to 60 pulses/s increases the speed but may increase risk of inadvertent injuries to adjacent vessel wall as the procedure is so quick and therefore difficult to control. In situ laser fenestration in the Zenith TX2 was also easily achieved within a reasonable time frame. For the C-TAG, the time taken to create fenestrations was too long in our study to make it clinically feasible. Performing a laser fenestration which takes longer than a few seconds may pose unnecessary clinical risk to the patient due to the risk of iatrogenic injury to surrounding structures if the laser is malpositioned. There is also a risk of delayed perfusion of vital organs. This is even more important when performing ISLF to treat catastrophic aortic pathologies.^14^ Furthermore, large fabric tears in several fenestrations in the C-TAG were noted when the ISF were dilated with an 8 mm balloon, and a tear occurred when deploying/flaring a VBX bridging stent-graft in one of the fenestrations of the C-TAG. These results are similar to the findings in a recently-published systematic review.^13^ In addition to the difficulties of making a fenestration in C-TAG (made from expanded polytetrafluoroethylene [ePTFE] fabric), there are previous reports of hazardous chemicals by-production when polytetrafluoroethylene (PTFE) and/or ePTFE is exposed to thermal energy.^15–17^ As a result, despite otherwise appealing features of the C-TAG in endovascular treatment of the thoracic aorta,^18^ the creation of ISLF by means of the 308 nm CVX-300 Excimer Laser System in C-TAG cannot be encouraged. It is likely the same situation with other PTFE grafts, but we did not test any other brand.

Surprisingly frequent and significant stenosis were noted when deploying and flaring bridging stent-grafts using nominal balloon pressure. Satisfactory deployment of the bridging stent-grafts was only noted when the stents were inflated and/or flared with high-pressure balloons. This was the case with both Zenith Alpha and Zenith TX2 endografts. The Zenith TX2 graft was most resistant to dilatation of the fenestration, and only with BeGraft peripheral stents, there were no residual stenoses left behind. Previously, Lin et al^19^ found that the fenestrations remained narrow when it was dilated with a 6 mm noncompliant balloon. They also noted fraying of the fabrics when large size balloons (≥10 mm) were used.^20^ Studies from Lin et al,^12^ however, suggest a theoretical advantage of using an energy-based method because the fusion of fibers created by this technique may prevent some of the fraying observed after balloon dilation.^19^

Although fenestrations produced by laser are unlikely to produce particles which may embolise,^21^ having a tight seal between the bridging stent-graft and the endograft not only reduces the risk for type IIIc endoleak^22^ but may also prevent embolisation.^23^ The authors Lin et al even go as far as stating that for ISLF, one should give preference to endografts with multifilament yarns such as Cook Zenith graft as the dispersed fibers may contribute to hemostasis at the edge of the fenestration and branch graft interface.^23^ In another study by Lin et al,^19^ some debris was produced by the ISLF technique. When the debris was examined after 25 fenestrations per graft, only a very small volume of debris was however noted. Given that the ISF technique is usually used to make no more than 4 fenestrations per patient, the authors claim that the debris load was very low and much less than expected.^19^

The authors Lin et al^19^ noted that in an ex vivo model, large balloons (ie, ≥10 mm) increased the destruction and tearing of polyester fabrics of the endografts. As a result, the maximum dilation that Lin et al^19^ recommended was 6 to 8 mm to avoid major tears. In our study protocol, the fenestrations were not dilated with a balloon greater than 8 mm to avoid major tears; however, when flaring the bridging stent-grafts with balloons ≥10 mm, no tears were noted in the Zenith Alpha and Zenith TX2 endografts. These results may suggest that having a bridging stent-graft in the ISF may be protective against major tear in the endograft.

### Limitations

Our endografts were tested in 37°C saline to mimic in vivo conditions. Despite this environment, endografts may behave differently when ISLF is carried out in patients. Furthermore, we did not test other types of bridging stent-grafts and/or commercially-available endografts. Thus, the results of this experiment cannot be generalized to other types of bridging stent-grafts and/or endografts. Also, our results are limited to the 308 nm CVX-300 Excimer Laser System and may not apply to other laser systems. Limited number of VBX stents were available for this experiment and as a result, this limits its evaluation. Evaluation of the stenoses were carried out by visual inspection, and therefore the clinical impact on hemoperfusion of vital organs and effect on the blood flow in the endograft and bridging stent-grafts cannot be evaluated from this study. One important limitation of this study is of course the short term of in vitro outcomes. As been discussed before, there is a question mark on whether there will be late graft tears and subsequent loss of integrity, over the lifetime of the patient when done in vivo.^24^

## Conclusion

Both Zenith Alpha and Zenith TX2 endografts responded well to the ISLF technique. C-TAG was difficult to fenestrate, and dilatation of the fenestrations was prone to fabric tears. Satisfactory bridging stent-graft deployment and elimination of stenoses were achieved when the bridging stent-grafts were flared with high-pressure balloons in the Zenith Alpha and Zenith TX2 endografts. Residual stenosis was more common in Zenith TX2 compared with Zenith Alpha, and best results were achieved together with BeGraft Peripheral. A small number of VBX bridging stent-grafts were available for this experiment, thus comparison with BeGraft Peripheral and BeGraft Peripheral Plus bridging stent-grafts is limited. Further work with different types of commercially-available bridging stent-grafts and endografts in ISLF in patients is needed to provide clinical outcomes as well.

## Supplemental Material

sj-docx-1-jet-10.1177_15266028221119315 – Supplemental material for In Situ Laser Fenestration Technique: Bench-Testing of Aortic Endograft to Guide Clinical Practice Click here for additional data file. Supplemental material, sj-docx-1-jet-10.1177_15266028221119315 for In Situ Laser Fenestration Technique: Bench-Testing of Aortic Endograft to Guide Clinical Practice by Matthew Joe Grima, Anders Wanhainen and David Lindström in Journal of Endovascular Therapy

sj-docx-2-jet-10.1177_15266028221119315 – Supplemental material for In Situ Laser Fenestration Technique: Bench-Testing of Aortic Endograft to Guide Clinical Practice Click here for additional data file. Supplemental material, sj-docx-2-jet-10.1177_15266028221119315 for In Situ Laser Fenestration Technique: Bench-Testing of Aortic Endograft to Guide Clinical Practice by Matthew Joe Grima, Anders Wanhainen and David Lindström in Journal of Endovascular Therapy

sj-jpg-6-jet-10.1177_15266028221119315 – Supplemental material for In Situ Laser Fenestration Technique: Bench-Testing of Aortic Endograft to Guide Clinical Practice Click here for additional data file. Supplemental material, sj-jpg-6-jet-10.1177_15266028221119315 for In Situ Laser Fenestration Technique: Bench-Testing of Aortic Endograft to Guide Clinical Practice by Matthew Joe Grima, Anders Wanhainen and David Lindström in Journal of Endovascular Therapy

sj-jpg-7-jet-10.1177_15266028221119315 – Supplemental material for In Situ Laser Fenestration Technique: Bench-Testing of Aortic Endograft to Guide Clinical Practice Click here for additional data file. Supplemental material, sj-jpg-7-jet-10.1177_15266028221119315 for In Situ Laser Fenestration Technique: Bench-Testing of Aortic Endograft to Guide Clinical Practice by Matthew Joe Grima, Anders Wanhainen and David Lindström in Journal of Endovascular Therapy

sj-pdf-3-jet-10.1177_15266028221119315 – Supplemental material for In Situ Laser Fenestration Technique: Bench-Testing of Aortic Endograft to Guide Clinical Practice Click here for additional data file. Supplemental material, sj-pdf-3-jet-10.1177_15266028221119315 for In Situ Laser Fenestration Technique: Bench-Testing of Aortic Endograft to Guide Clinical Practice by Matthew Joe Grima, Anders Wanhainen and David Lindström in Journal of Endovascular Therapy

sj-pdf-4-jet-10.1177_15266028221119315 – Supplemental material for In Situ Laser Fenestration Technique: Bench-Testing of Aortic Endograft to Guide Clinical Practice Click here for additional data file. Supplemental material, sj-pdf-4-jet-10.1177_15266028221119315 for In Situ Laser Fenestration Technique: Bench-Testing of Aortic Endograft to Guide Clinical Practice by Matthew Joe Grima, Anders Wanhainen and David Lindström in Journal of Endovascular Therapy

sj-pdf-5-jet-10.1177_15266028221119315 – Supplemental material for In Situ Laser Fenestration Technique: Bench-Testing of Aortic Endograft to Guide Clinical Practice Click here for additional data file. Supplemental material, sj-pdf-5-jet-10.1177_15266028221119315 for In Situ Laser Fenestration Technique: Bench-Testing of Aortic Endograft to Guide Clinical Practice by Matthew Joe Grima, Anders Wanhainen and David Lindström in Journal of Endovascular Therapy
