# Supplementary material for: In Situ Laser Fenestration Technique: Bench-Testing of Aortic Endograft to Guide Clinical Practice
Source: J Endovasc Ther. 2022 Aug 24;31(1):126–31. doi: 10.1177/15266028221119315 (PMC10773159; doi:10.1177/15266028221119315)
Supplement: sj-docx-1-jet-10.1177_15266028221119315 – Supplemental material for In Situ Laser Fenestration Technique: Bench-Testing of Aortic Endograft to Guide Clinical Practice [file sj-docx-1-jet-10.1177_15266028221119315.docx]

**Supplementary Figure 1:**

Setup of the experiment
