# Supplementary material for: In Situ Laser Fenestration Technique: Bench-Testing of Aortic Endograft to Guide Clinical Practice
Source: J Endovasc Ther. 2022 Aug 24;31(1):126–31. doi: 10.1177/15266028221119315 (PMC10773159; doi:10.1177/15266028221119315)
Supplement: sj-docx-2-jet-10.1177_15266028221119315 – Supplemental material for In Situ Laser Fenestration Technique: Bench-Testing of Aortic Endograft to Guide Clinical Practice [file sj-docx-2-jet-10.1177_15266028221119315.docx]

**Supplementary Table 1:**

Time (s) to create ISLF

| Experiment I.i - Time in seconds (s) | |
| --- | --- |
| *Fluency 30 mJ/mm^2^ and Rate 25 pulses/second* | |
| **Zenith Alpha™** | **Zenith TX2®** |
| 1.6 | 1.7 |
| 2.0 | 1.8 |
| 1.9 | 2.1 |
| 1.6 | 1.6 |
| 1.6 | 1.6 |
| 1.5 | 1.4 |
| 1.8 | 1.8 |
| 1.5 | 2.0 |
| 1.4 | 2.4 |
| 1.6 | 1.6 |

|  |  |
| --- | --- |
| Experiment I.ii - Time in seconds (s) | |
| *Fluency 45 mJ/mm^2^ and Rate 25 pulses/second* | |
| **Zenith Alpha™** | **Zenith TX2®** |
| 0.9 | 1.1 |
| 1.0 | 1.0 |
| 0.7 | 1.6 |
| 0.6 | 1.1 |
| 0.9 | 1.4 |
| 0.9 | 1.2 |
| 0.8 | 1.3 |
| 1.0 | 1.3 |
| 0.7 | 1.3 |
| 0.9 | 1.2 |
|  |  |

| Experiment I.iii - Time in seconds (s) | |
| --- | --- |
| *Fluency 45 mJ/mm^2^ and Rate 60 pulses/second* | |
| **Zenith TX2®** | **C-TAG (no lamellae)** |
| 0.4 | 3.2 |
| 0.5 | 7.3 |
| 0.5 | 9.0 |
| 0.6 | 7.5 |
| 0.4 | 17.1 |
| 0.4 | 15.3 |
| 0.6 | 9.7 |
| 0.6 | 8.3 |
| 0.5 | 8.8 |
| 0.4 | 17.0 |
