# Supplementary figures and images for: In Situ Laser Fenestration Technique: Bench-Testing of Aortic Endograft to Guide Clinical Practice
Source: J Endovasc Ther. 2022 Aug 24;31(1):126–31. doi: 10.1177/15266028221119315 (PMC10773159; doi:10.1177/15266028221119315)

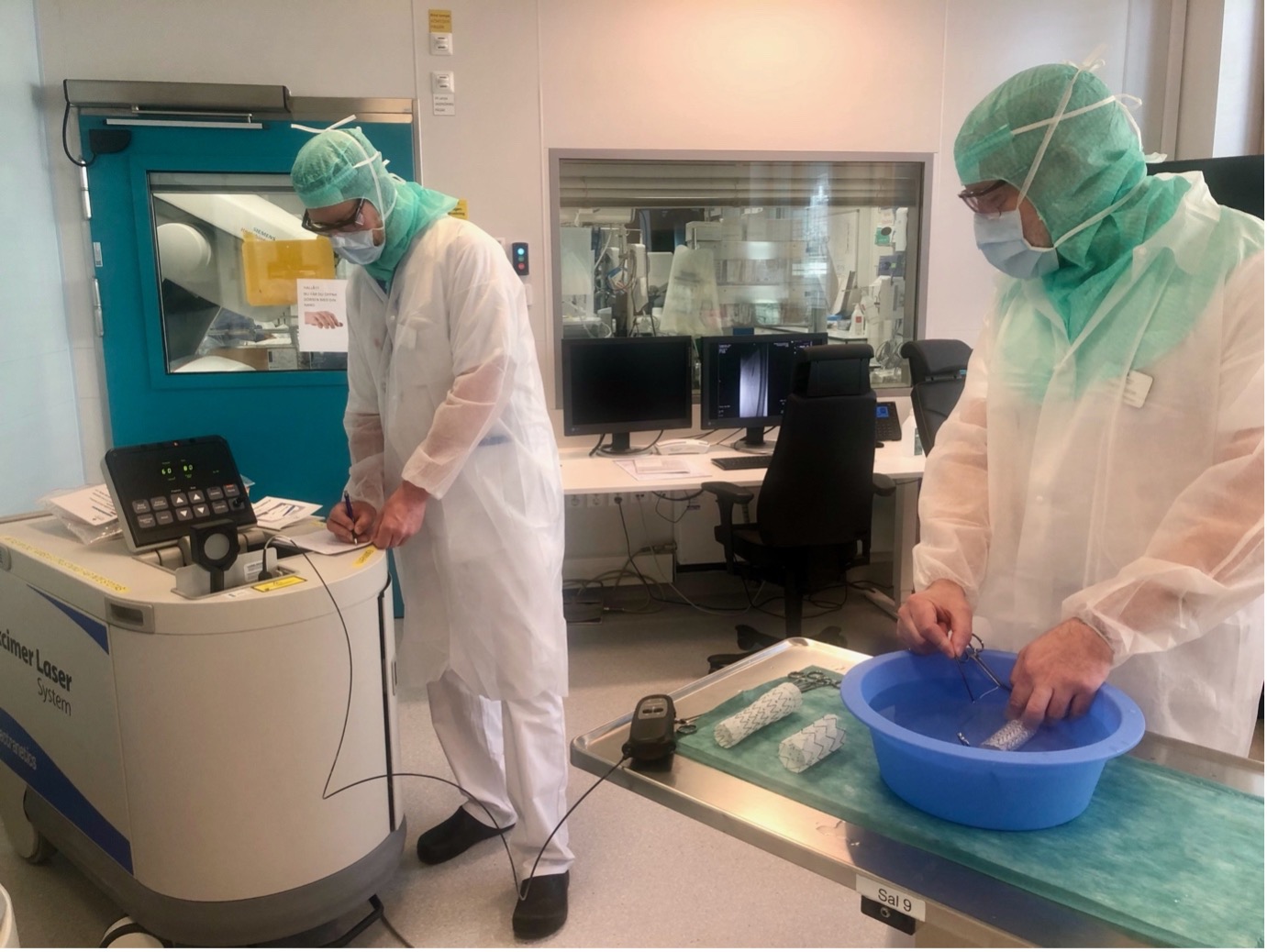

Supplement: sj-jpg-6-jet-10.1177_15266028221119315 – Supplemental material for In Situ Laser Fenestration Technique: Bench-Testing of Aortic Endograft to Guide Clinical Practice [file sj-jpg-6-jet-10.1177_15266028221119315.jpg]

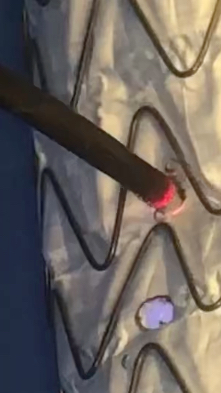

Supplement: sj-jpg-7-jet-10.1177_15266028221119315 – Supplemental material for In Situ Laser Fenestration Technique: Bench-Testing of Aortic Endograft to Guide Clinical Practice [file sj-jpg-7-jet-10.1177_15266028221119315.jpg]
