# Supplementary material for: In Situ Laser Fenestration Technique: Bench-Testing of Aortic Endograft to Guide Clinical Practice
Source: J Endovasc Ther. 2022 Aug 24;31(1):126–31. doi: 10.1177/15266028221119315 (PMC10773159; doi:10.1177/15266028221119315)
Supplement: sj-pdf-3-jet-10.1177_15266028221119315 – Supplemental material for In Situ Laser Fenestration Technique: Bench-Testing of Aortic Endograft to Guide Clinical Practice [file sj-pdf-3-jet-10.1177_15266028221119315.pdf]

Supplementary table 3: Response of BeGraft Peripheral in ISF

Zenith Alpha™				
**Bridging stent-graft**	**Experiment III.i: Bridging stent-grafts deployed at nominal pressure of 8 atmospheres**	**Experiment III.ii: Bridging stent-grafts flared with Armada™ 10x20mm balloon at nominal pressure of 6 atmospheres to mimic renal artery stenting.**	**Experiment III.iii: Bridging stent-grafts flared with Armada™ 12x20mm balloon at nominal pressure of 4 atmospheres to mimic a superior mesenteric artery stenting**	**Experiment III.iv: Bridging stent-grafts flared with Armada™ 12x20mm balloon at 10 atmospheres to simulate a higher-pressure balloon.**
**1.BeGraft 8x37mm**	No rupture/No spin/**minimal stenosis**	No rupture/No spin/**significant stenosis**	No rupture/No spin/**minimal stenosis**	No rupture/No spin /No stenosis
**2.BeGraft 8x37mm**	No rupture/No spin/**significant stenosis**	No rupture/No spin/**minimal stenosis**	No rupture/No spin/**minimal stenosis**	No rupture/No spin/ No stenosis
**3. BeGraft 8x37mm**	No rupture/No spin/**significant stenosis**	No rupture/No spin/**minimal stenosis**	No rupture/No spin/**minimal stenosis**	No rupture/No spin/ No stenosis
**4. BeGraft 8x37mm**	No rupture/No spin/**minimal stenosis**	No rupture/No spin/**significant stenosis**	No rupture/No spin/**significant stenosis**	No rupture/No spin/ No stenosis

**5. BeGraft 8x37mm**	No rupture/No spin/**significant stenosis**	No rupture/No spin/**minimal stenosis**	No rupture/No spin/**significant stenosis**	No rupture/No spin/ No stenosis
**6. BeGraft 8x37mm**	No rupture/No spin/**significant stenosis**	No rupture/No spin/**significant stenosis**	No rupture/No spin/**significant stenosis**	No rupture/No spin/ No stenosis
**7. BeGraft 8x37mm**	No rupture/No spin/**significant stenosis**	No rupture/No spin/**minimal stenosis**	No rupture/No spin/**significant stenosis**	No rupture/No spin/ No stenosis
**8. BeGraft 8x37mm**	No rupture/No spin/**significant stenosis**	No rupture/No spin/**significant stenosis**	No rupture/No spin/**significant stenosis**	No rupture/No spin/ No stenosis
**9. BeGraft 8x37mm**	No rupture/No spin/**significant stenosis**	No rupture/No spin/**significant stenosis**	No rupture/No spin/**significant stenosis**	No rupture/No spin/ No stenosis
**10. BeGraft 8x37mm**	No rupture/No spin/**minimal stenosis**	No rupture/No spin/**significant stenosis**	No rupture/No spin/**significant stenosis**	No rupture/No spin/ No stenosis

**Zenith TX2™**				
**Bridging stent-graft**	**Experiment III.i Bridging stent-grafts deployed at nominal pressure of 8 atmospheres**	**Experiment III.ii: Bridging stent-grafts flared with Armada™ 10x20mm balloon at nominal pressure of 6 atmospheres to mimic renal artery stenting.**	**Experiment III.iii: Bridging stent-grafts flared with Armada™ 12x20mm balloon at nominal pressure of 4 atmospheres to mimic a superior mesenteric artery stenting**	**Experiment III.iv: Bridging stent-grafts flared with Armada™ 12x20mm balloon at 10 atmospheres to simulate a higher-pressure balloon.**
**1.BeGraft 8x37mm**	No rupture/No spin/**significant stenosis**	No rupture/No spin/**significant stenosis**	No rupture/No spin/**significant stenosis**	No rupture/No spin/ **minimal stenosis**
**2.BeGraft 8x37mm**	No rupture/No spin/**significant stenosis**	No rupture/No spin/**significant stenosis**	No rupture/No spin/**significant stenosis**	No rupture/No spin/no stenosis
**3. BeGraft 8x37mm**	No rupture/No spin/**significant stenosis**	No rupture/No spin/**significant stenosis**	No rupture/No spin/**significant stenosis**	No rupture/No spin/no stenosis
**4. BeGraft 8x37mm**	No rupture/No spin/**significant stenosis**	No rupture/No spin/**significant stenosis**	No rupture/No spin/**significant stenosis**	No rupture/No spin/no stenosis

**5. BeGraft 8x37mm**	No rupture/No spin/**significant stenosis**	No rupture/No spin/**significant stenosis**	No rupture/No spin/**significant stenosis**	No rupture/No spin/no stenosis
**6. BeGraft 8x37mm**	No rupture/No spin/**significant stenosis**	No rupture/No spin/**significant stenosis**	No rupture/No spin/**significant stenosis**	No rupture/No spin/no stenosis
**7. BeGraft 8x37mm**	No rupture/No spin/**significant stenosis**	No rupture/No spin/**significant stenosis**	No rupture/No spin/**significant stenosis**	No rupture/No spin/**minimal stenosis**
**8. BeGraft 8x37mm**	No rupture/No spin/**significant stenosis**	No rupture/No spin/**significant stenosis**	No rupture/No spin/**significant stenosis**	No rupture/No spin/no stenosis
**9. BeGraft 8x37mm**	No rupture/No spin/**significant stenosis**	No rupture/No spin/**significant stenosis**	No rupture/No spin/**significant stenosis**	No rupture/No spin/no stenosis
**10. BeGraft 8x37mm**	No rupture/No spin/sig**nificant stenosis**	No rupture/No spin/**significant stenosis**	No rupture/No spin/**significant stenosis**	No rupture/No spin/no stenosis
