# Supplementary material for: In Situ Laser Fenestration Technique: Bench-Testing of Aortic Endograft to Guide Clinical Practice
Source: J Endovasc Ther. 2022 Aug 24;31(1):126–31. doi: 10.1177/15266028221119315 (PMC10773159; doi:10.1177/15266028221119315)
Supplement: sj-pdf-4-jet-10.1177_15266028221119315 – Supplemental material for In Situ Laser Fenestration Technique: Bench-Testing of Aortic Endograft to Guide Clinical Practice [file sj-pdf-4-jet-10.1177_15266028221119315.pdf]

**Supplementary table 4: Response of BeGraft Peripheral plus in ISF**

| Zenith Alpha™ | | | | |
|---|---|---|---|---|
| **Bridging stent-graft** | **Experiment III.i Bridging stent-graft deployed at nominal pressure of 9 atmospheres** | **Experiment III.ii: Bridging stent-grafts flared with Armada™ 10x20mm balloon at nominal pressure of 6 atmospheres to mimic renal artery stenting.** | **Experiment III.iii: Bridging stent-grafts flared with Armada™ 12x20mm balloon at nominal pressure of 4 atmospheres to mimic a superior mesenteric artery stenting** | **Experiment III.iv: Bridging stent-grafts flared with Armada™ 12x20mm balloon at 10 atmospheres to simulate a higher-pressure balloon.** |
| **1.BeGraft plus 8x37mm** | No rupture/No spin/**minimal stenosis** | No rupture/No spin/No stenosis | No rupture/No spin/ No stenosis | No rupture/No spin/ No stenosis |
| **2.BeGraft plus 8x37mm** | No rupture/No spin/ **minimal stenosis** | No rupture/No spin/ **significant stenosis** | No rupture/No spin/ **significant stenosis** | No rupture/No spin/ No stenosis |
| **3. BeGraft plus 8x37mm** | No rupture/No spin/**significant stenosis** | No rupture/No spin/ **significant stenosis** | No rupture/No spin/ **significant stenosis** | No rupture/No spin/ No stenosis |

| | | | | |
|---|---|---|---|---|
| **4. BeGraft plus 8x37mm** | No rupture/No spin/ **significant stenosis** | No rupture/No spin/**significant stenosis** | No rupture/No spin/**significant stenosis** | No rupture/No spin/ No stenosis |
| **5. BeGraft plus 8x37mm** | No rupture/No spin/**significant stenosis** | No rupture/No spin/ **significant stenosis** | No rupture/No spin/**significant stenosis** | No rupture/No spin/ No stenosis |
| **6. BeGraft plus 8x37mm** | No rupture/No spin/**significant stenosis** | No rupture/No spin/**significant stenosis** | No rupture/No spin/**significant stenosis** | No rupture/No spin/ No stenosis |
| **7. BeGraft plus 8x37mm** | No rupture/No spin/**significant stenosis** | No rupture/No spin/ **significant stenosis** | No rupture/No spin/**significant stenosis** | No rupture/No spin/ No stenosis |
| **8. BeGraft plus 8x37mm** | No rupture/No spin/**significant stenosis** | No rupture/No spin/**significant stenosis** | No rupture/No spin/**significant stenosis** | No rupture/No spin/ No stenosis |
| **9. BeGraft plus 8x37mm** | No rupture/No spin/**significant stenosis** | No rupture/No spin/**significant stenosis** | No rupture/No spin/**significant stenosis** | No rupture/No spin/ No stenosis |
| **10. BeGraft plus 8x37mm** | No rupture/No spin/ **significant stenosis** | No rupture/No spin/**significant stenosis** | No rupture/No spin/**significant stenosis** | No rupture/No spin/ No stenosis |

| **Zenith TX2** | | | | |
|---|---|---|---|---|
| **Bridging stent-graft** | **Experiment III.i Bridging stent-graft deployed at nominal pressure of 9 atmospheres** | **Experiment III.ii: Bridging stent-grafts flared with Armada™ 10x20mm balloon at nominal pressure of 6 atmospheres to mimic renal artery stenting.** | **Experiment III.iii: Bridging stent-grafts flared with Armada™ 12x20mm balloon at nominal pressure of 4 atmospheres to mimic a superior mesenteric artery stenting** | **Experiment III.iv: Bridging stent-grafts flared with Armada™ 12x20mm balloon at 10 atmospheres to simulate a higher-pressure balloon.** |
| **1.BeGraft plus 8x37mm** | No rupture/No spin/ **significant stenosis** | No rupture/No spin/ **significant stenosis** | No rupture/No spin/ **significant stenosis** | No rupture/No spin/ **significant stenosis** |
| **2.BeGraft plus 8x37mm** | No rupture/No spin/ **significant stenosis** | No rupture/No spin/ **significant stenosis** | No rupture/No spin/ **significant stenosis** | No rupture/No spin/ **significant stenosis** |
| **3. BeGraft plus 8x37mm** | No rupture/No spin/**significant stenosis** | No rupture/No spin/ **significant stenosis** | No rupture/No spin/ **significant stenosis** | No rupture/No spin/ **significant stenosis** |
| **4. BeGraft plus 8x37mm** | No rupture/No spin/ **significant stenosis** | No rupture/No spin/ **significant stenosis** | No rupture/No spin/ **significant stenosis** | No rupture/No spin/ **significant stenosis** |
| **5. BeGraft plus 8x37mm** | No rupture/No spin/**significant stenosis** | No rupture/No spin/ **significant stenosis** | No rupture/No spin/ **significant stenosis** | No rupture/No spin/ **significant stenosis** |

| | | | | |
|---|---|---|---|---|
| **6. BeGraft plus 8x37mm** | No rupture/No spin/**significant stenosis** | No rupture/No spin/ **significant stenosis** | No rupture/No spin/ **significant stenosis** | No rupture/No spin/ **significant stenosis** |
| **7. BeGraft plus 8x37mm** | No rupture/No spin/**significant stenosis** | No rupture/No spin/ **significant stenosis** | No rupture/No spin/ **significant stenosis** | No rupture/No spin/ **significant stenosis** |
| **8. BeGraft plus 8x37mm** | No rupture/No spin/**significant stenosis** | No rupture/No spin/ **significant stenosis** | No rupture/No spin/ **significant stenosis** | No rupture/No spin/ **significant stenosis** |
| **9. BeGraft plus 8x37mm** | No rupture/No spin/**significant stenosis** | No rupture/No spin/ **significant stenosis** | No rupture/No spin/ **significant stenosis** | No rupture/No spin/ **significant stenosis** |
| **10. BeGraft plus 8x37mm** | No rupture/No spin/ **significant stenosis** | No rupture/No spin/ **significant stenosis** | No rupture/No spin/ **significant stenosis** | No rupture/No spin/ **significant stenosis** |
