# Supplementary material for: In Situ Laser Fenestration Technique: Bench-Testing of Aortic Endograft to Guide Clinical Practice
Source: J Endovasc Ther. 2022 Aug 24;31(1):126–31. doi: 10.1177/15266028221119315 (PMC10773159; doi:10.1177/15266028221119315)
Supplement: sj-pdf-5-jet-10.1177_15266028221119315 – Supplemental material for In Situ Laser Fenestration Technique: Bench-Testing of Aortic Endograft to Guide Clinical Practice [file sj-pdf-5-jet-10.1177_15266028221119315.pdf]

Supplementary table 2: Response of GORE® VIABAHN® VBX Balloon Expandable Endoprosthesis in ISF

Zenith Alpha™				
**Bridging stent-graft**	**Experiment III.i: Bridging Stent-grafts deployed at nominal pressure of 12 atmospheres**	**Experiment III.ii: Bridging stent-grafts flared with Armada™ 10x20mm balloon at nominal pressure of 6 atmospheres to mimic renal artery stenting.**	**Experiment III.iii: Bridging stent-grafts flared with Armada™ 12x20mm balloon at nominal pressure of 4 atmospheres to mimic a superior mesenteric artery stenting**	**Experiment III.iv: Bridging stent-grafts flared with Armada™ 12x20mm balloon at 10 atmospheres to simulate a higher-pressure balloon.**
1.VBX Bridging stent-graft 8mm x 39mm	No rupture / No stenosis / No spin	No rupture / **minimal stenosis** / No spin	No rupture / **minimal stenosis** / No spin	No rupture / no stenosis / No spin
2. VBX Bridging stent-graft 8mm x 39mm	No rupture / **minimal stenosis** / spins	No rupture / **minimal stenosis** / spins	No rupture / **minimal stenosis** / spins	No rupture / no stenosis / No spin / **Limited flaring**

3. VBX Bridging stent-graft 8mm x 39mm	No rupture / No stenosis / No spin	No rupture / **minimal stenosis** / No spin	No rupture / **minimal stenosis** / No spin	No rupture / no stenosis / No spin / **Limited flaring**

**Zenith TX2®**						
**Bridging stent-graft**	**Experiment III.i Bridging stent-graft deployed at nominal pressure of 12 atmospheres**	**Experiment III.ii: Bridging stent-graft flared with Armada™ 10x20mm balloon at nominal pressure of 6 atmospheres to mimic renal artery stenting.**	**Experiment III.iii: Bridging stent-graft flared with Armada™ 12x20mm balloon at nominal pressure of 4 atmospheres to mimic a superior mesenteric artery stenting**	**Experiment III.iv: Bridging stent-graft flared with Armada™ 12x20mm balloon at 10 atmospheres to simulate a higher-pressure balloon.**	**Bridging stent-graft flared with 10mm Armada™ balloon at 13 atmospheres.**	**Bridging stent-graft flared with Atlas™ GOLD Balloon 12 x 20mm at nominal pressure of 6 atmospheres and 18 atmospheres rated burst pressure.**
1. VBX Bridging stent-graft 8mm x 39mm	No rupture / **Significant stenosis** / No spin	No rupture / **Significant stenosis** / No spin	No rupture / **Significant stenosis** / No spin	No rupture / **minimal stenosis** / No spin	No stenosis / No spin	(At 11atmospheres) No stenosis / No spin

2. VBX Bridging stent-graft 8mm x 39mm	No rupture / **Significant stenosis** / No spin	No rupture / **Significant stenosis** / No spin	No rupture / **Significant stenosis** / No spin	No rupture / **Significant stenosis** / No spin	**Significant stenosis** / No spin	(At 11atmospheres) No stenosis / No spin
3. VBX Bridging stent-graft 8Lmm x39mm	No rupture / **Significant stenosis** / No spin	No rupture / **Significant stenosis** / spins	No rupture / **Significant stenosis** / No spin	No rupture / **Significant stenosis** / No spin	**Significant stenosis** / No spin	(At 11atmospheres) No stenosis / No spin

C-TAG				
**Bridging stent-graft**	**Experiment III.i Bridging stent-graft deployed at nominal pressure of 12 atmospheres**	**Experiment III.ii: The bridging stent-grafts were flared with Armada™ 10x20mm balloon at nominal pressure of 6 atmospheres to mimic renal artery stenting.**	**Experiment III.iii: The bridging stent-grafts were flared with Armada™ 12x20mm balloon at nominal pressure of 4 atmospheres to mimic a superior mesenteric artery stenting**	**Experiment III.iv: The bridging stent-grafts were flared with Armada™ 12x20mm balloon at 10 atmospheres to simulate a higher-pressure balloon.**
1. VBX Bridging stent-graft 8Lmm x 29mm	No rupture / no stenosis / Spins	No rupture / Minimal narrowing / Spins	No rupture / Minimal narrowing / spins	No rupture / Minimal narrowing / spins
2. VBX Bridging stent-graft 8mm x 29mm	C-TAG rupture* / no stenosis / Spins	No rupture / No stenosis / spins	No rupture / Minimal narrowing / Doesn't spin	C-TAG Ruptures*
3. VBX Bridging stent-graft 8mm x 39mm	No rupture / no stenosis / Spins	No rupture / Minimal narrowing / spins	No rupture / Minimal narrowing / spins	No rupture / No stenosis / Doesn't spin
